# The regulation of CPNE1 ubiquitination by the NEDD4L is involved in the pathogenesis of non-small cell lung cancer

**DOI:** 10.1038/s41420-021-00736-1

**Published:** 2021-11-06

**Authors:** Ruochen Zhang, Weijie Zhang, Yuanyuan Zeng, Yue Li, Jieqi Zhou, Yang Zhang, Anqi Wang, Yantian Lv, Jianjie Zhu, Zeyi Liu, Jian-an Huang

**Affiliations:** 1 Department of Pulmonary and Critical Care Medicine, the First Affiliated Hospital of Soochow University, Suzhou, 215006 China; 2 Institute of Respiratory Diseases, Soochow University, Suzhou, 215006 China; 3 Suzhou Key Laboratory for Respiratory Diseases, Suzhou, 215006 China

**Keywords:** Non-small-cell lung cancer, Proteomics

## Abstract

Our previous studies revealed that oncogene CPNE1 is positively correlated with the occurrence, TNM stage, lymph node metastasis, and distant metastasis of non-small-cell lung cancer (NSCLC), and it could be regulated by micro RNAs. But no direct role of post-translational modification of CPNE1 in NSCLC has been reported. This study confirms that CPNE1 is degraded by two pathways: the ubiquitin-proteasome pathway and the autophagy-lysosome pathway. CPNE1 binds with the ubiquitin molecule via its K157 residue. Moreover, we determined that the ubiquitin ligase NEDD4L can mediate the ubiquitination of CPNE1 and promote its degradation. In addition, we find that NEDD4L knockdown promotes the proliferation and metastasis of NSCLC cells by regulating CPNE1 in vitro and vivo. This study aims to further investigate the mechanism of CPNE1 ubiquitination in the occurrence and development of NSCLC and provide a new potential target for NSCLC treatment.

## Introduction

Lung cancer is the leading cause of death from malignancies worldwide [1]. NSCLC is characterized by difficulty in surgical resection, a tendency toward postoperative metastasis and recurrence, and insensitivity to radiotherapy and chemotherapy [2]. Traditional treatment regimens are not easy to undergo, which causes great distress to patients with non-small-cell lung cancer. In the past two decades, significant advances have been made in the treatment of NSCLC that have improved our understanding of the biology and pathogenesis of tumor disease. The use of small molecule tyrosine kinase inhibitors and immunotherapy can increase the survival benefit in lung cancer patients, but drug resistance is a problem in some patients after treatment [3, 4]. Currently, the overall cure and survival rates of NSCLC remain low. Therefore, it is of great importance to further study the specific molecular mechanism underlying the occurrence and development of lung cancer, search for new drug targets, reduce the mortality rate, and improve the prognosis of NSCLC patients.

Copine(CPNE) proteins, members of a family of calcium-dependent phospholipid-binding proteins, are highly conserved with high sequence similarity. They are widely distributed from plants to humans [5, 6]. The CPNE family contains nine members (CPNE1 to CPNE9), all of which have 2 tandem C2 domains (C2A and C2B) at the N-terminus and an A domain at the C-terminus [7]. The C2 domains, which have been originally identified in protein kinase C [8], are thought to play important roles in signal transduction and membrane trafficking [9]. The A-domain, named for von Willebrand factor, plasma and extracellular matrix protein, has been studied in integrins and several extracellular matrix proteins and appears to function as a protein-binding domain [8, 10]. This family of proteins regulates multiple biological functions, including cell proliferation, migration, and differentiation [11–13].

High expression of CPNE1 was correlated with poor prognosis and clinical progression in various cancers. Evidence implies that high CPNE1 expression might be an independent prognostic indicator of poor recurrence-free survival in prostate cancer [14]. In addition, knockdown of CPNE1 inhibits osteosarcoma progression by downregulating the expression of Ras, MEK-1/2, WNT1, β-catenin, cyclin A1, IRAK2, and cIAP2 [15]. In triple-negative breast cancer, CPNE1 induces tumorigenesis and radio resistance by activating the AKT pathway [16]. Moreover, 14-3-3γ and Jab1 induce CPNE1-mediated neuronal differentiation by directly binding to CPNE1 [17, 18]. Conversely, HAX1 has an opposing effect [19]. Our previous study found that high expression of CPNE1 was positively correlated with TNM stage, lymph node metastasis, and distant metastasis in 128 lung cancer tissues. In addition, CPNE1 could be negatively regulated by several micro RNAs [20, 21].

Neural precursor cell expressed developmentally downregulated 4-like (NEDD4L) is a member of the NEDD4 family and contains a C2 domain for membrane binding, a HECT domain for Ub protein ligation, and a central region including 4 WW domains for substrate recognition [22, 23]. NEDD4L was downregulated in NSCLCs. This downregulation correlated with lymph node invasion, advanced stage and poor survival [24]. As a ubiquitin ligase, NEDD4L specifically identifies a TGF-β-induced phospho-Thr-Pro-Tyr motif, inducing Smad2/3 polyubiquitination and degradation [25, 26]. In melanoma, NEDD4L is activated by p-MEK1/2 and ubiquitinates SP1 at the K685 residue, which leads to proteasomal degradation of SP1 [27]. NEDD4L acts as a tumor suppressor gene and is downregulated in lung cancer, indicating that it has a role in the initiation and progression of lung cancer [28].

Although the regulation of CPNE1 at transcriptional level has been extensively studied, we explored CPNE1 degradation via the ubiquitin-proteasome pathway and revealed that NEDD4L directly interacted with CPNE1 as a ubiquitin ligase. Furthermore, the underlying mechanism was elucidated.

## Results

### CPNE1 is elevated in lung cancer and correlates with poor survival

**Fig. 1:**
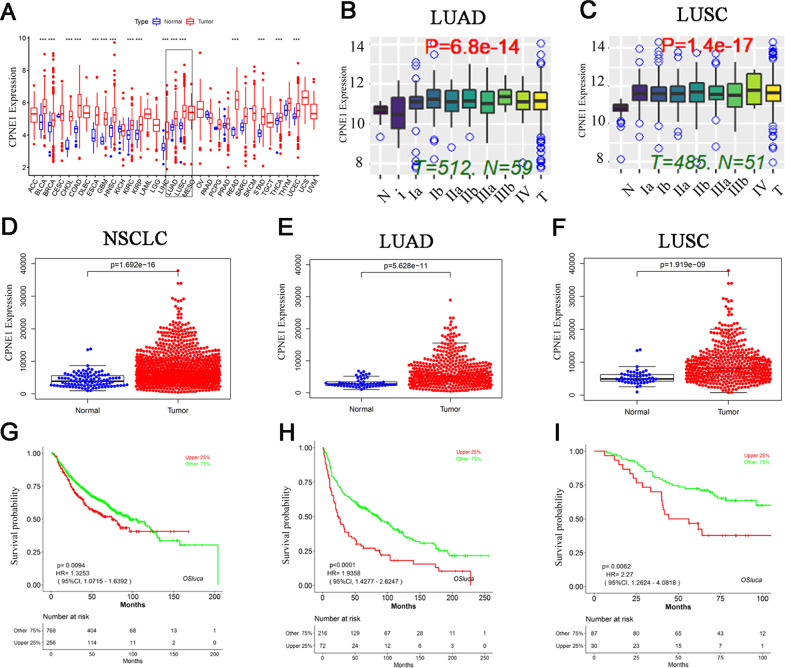
CPNE1 is elevated in lung cancer and correlates with poor survival. **A** CPNE1 is upregulated in a variety of cancers. **B**, **C** CPNE1 expression is correlated with the TNM stage of patients with lung adenocarcinoma/lung squamous cell carcinoma. *P* < 0.05 indicates that a difference of CPNE1 between groups (tumor and normal) was significant. **D**–**F** CPNE1 is overexpressed in NSCLC. **G**–**I** CPNE1 expression is correlated with poor survival in lung adenocarcinoma/lung squamous cell carcinoma. All statistical analysis applied in the results are Student’s *t* test.

We first investigated the possible role of CPNE1 by analyzing data in a publicly available database. TCGA database showed that CPNE1 is upregulated in a variety of cancers (https://portal.gdc.cancer.gov) (Fig. 1A). We found that CPNE1 is an oncogene in the OSluca database (http://bioinfo.henu.edu.cn) (Fig. S1A) [29]. In addition, high expression of CPNE1 was related to the TNM stage of patients with lung adenocarcinoma and lung squamous cell carcinoma (https://ccsm.uth.edu/miRacDB) (Fig.1B, C). Then, analysis of the TCGA database showed that CPNE1 was highly expressed in NSCLC (Fig. 1D), both in lung adenocarcinoma and lung squamous cell carcinoma (Fig. 1E, F). Analysis of survival data showed that high expression of CPNE1 was correlated with poor survival in lung cancer patients in three different databases (http://bioinfo.henu.edu.cn/LUCA/LUCAList.jsp) (Fig.1G–I).

### CPNE1 is degraded through both the ubiquitin-proteasome pathway and the autophagy-lysosome pathway

**Fig. 2:**
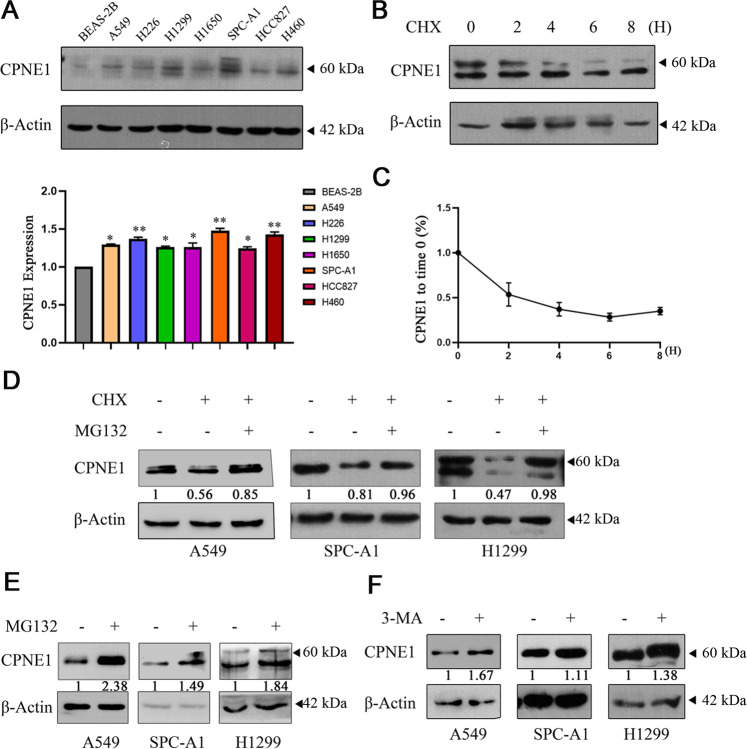
CPNE1 is degraded through both the ubiquitin-proteasome pathway and the autophagy-lysosome pathway. **A** CPNE1 was highly expressed in lung cancer cell lines. **B**, **C** CPNE1 was rapidly degraded in HEK293T cells. HEK293T cells were transfected with the Flag-CPNE1 plasmid. After 48 h, cells were treated with 100 µg/ml cycloheximide/vehicle for the indicated times. Cell lysates were prepared for Western blot analysis. The relative density of the CPNE1 band was measured with ImageJ software. **D** The degradation of CPNE1 mediated by CHX was rescued by MG132. The indicated cell lines were seeded into six-well plates. After 24 h, cells were treated with 100 µg/ml CHX or 100 µg/ml CHX plus 10 µM MG132 for 6 h. Cell lysates were prepared for Western blot analysis. **E**, **F** Both MG132 and 3-MA can stabilize CPNE1. The indicated cell lines were seeded into six-well plates. After 24 h, cells were treated with 10 µM MG132 for 6 h and 1 mM 3-MA for 18 h. Cell lysates were prepared for Western blot analysis.

We found that the expression of CPNE1 in lung cancer cell lines (A549, H226, H1299, H1650, SPC-A1, HCC827, and H460) was higher than that in a normal lung epithelial cell line (BEAS-2B) using Western blotting and qRT-PCR (Fig. 2A). This result was consistent with previous conclusions obtained by database analysis. We next measured CPNE1 degradation rates by a cycloheximide (CHX) chase assay. As shown in Fig. 2B, CHX treatment led to CPNE1 protein degradation in a time-dependent manner in HEK293T cells. In addition, the CPNE1 protein level was significantly reduced after CHX treatment for 2 h (Fig. 2B, C). This result confirms that the CPNE1 protein is unstable, suggesting that analyzing the mechanism of CPNE1 protein degradation is of great importance for the functional study of CPNE1. To determine whether this degradation involves the proteasome pathway, we treated lung cancer cells with CHX alone or with both CHX and MG132 to inhibit proteasomal degradation (Fig. 2D). In the absence of MG132, CPNE1 was degraded, while proteasome inhibition by MG132 reversed this degradation, notably stabilizing CPNE1. The above results indicated that degradation of the CPNE1 protein occurred through a proteasome-mediated ubiquitination degradation pathway. Interestingly, to further investigate the degradation of the CPNE1 protein, we evaluated the degradation of endogenous CPNE1 through the lysosomal pathway and the proteasome pathway. The results showed that when MG132 or 3-MA was added, endogenous CPNE1 protein accumulated (Fig. 2E, F). Taken together, these results confirm that the CPNE1 protein is degraded by both the ubiquitin-proteasome pathway and the lysosomal pathway in lung cancer cells.

### CPNE1 undergoes ubiquitination, and a highly conserved lysine at position 157 is subjected to ubiquitination

**Fig. 3:**
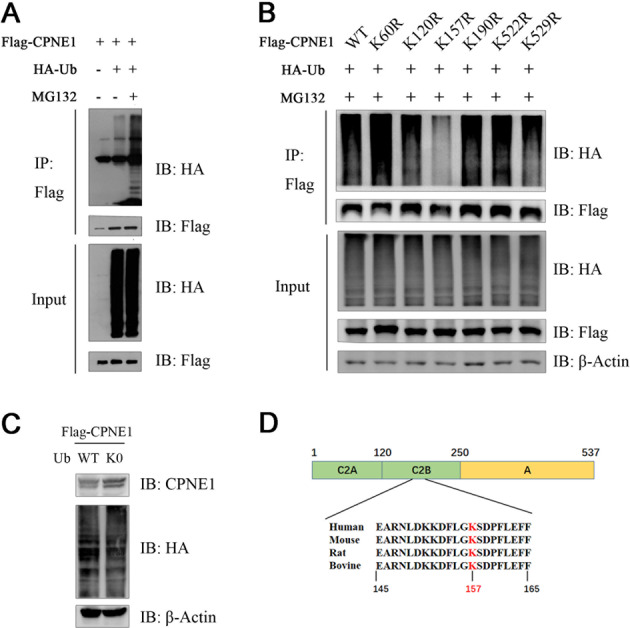
CPNE1 undergoes ubiquitination, and a highly conserved lysine at position 157 is subjected to ubiquitination. **A** The co-IP assay confirmed the association between exogenous CPNE1 and Ub in HEK293T cells. HEK293T cells were transfected with 2 µg of the Flag-CPNE1 plasmid and 2 µg of the HA-Ub plasmid. After 24 h, cells were harvested with RIPA lysis buffer. Co-IP was performed as indicated. **B** Co-expression with Ub-K0 increased the expression of CPNE1. HEK293T cells were transfected with the Flag-CPNE1 plasmid and HA-Ub-WT/HA-Ub-K0 plasmids. Cell lysates were prepared for Western blot analysis. **C** The K157 residue is the site of the interaction between CPNE1 and Ub. HEK293T cells were transfected with 2 µg of the Flag-CPNE1-WT/mutant plasmids and 2 µg of the HA-Ub plasmid. MG132 was added 2 h before cell harvesting. Co-IP was performed as indicated. **D** The CPNE1 lysine 157 residue is conserved in many species, including humans, mice, rats, and bovines. The conserved lysine residue is indicated in red.

To gain further insight into the mechanism of CPNE1 degradation, we evaluated whether CPNE1 is conjugated with Ub in vivo. Ubiquitin (Ub) and Flag-CPNE1 plasmids were co-transformed into HEK293T cells. The results showed obvious ubiquitin bands, and MG132 strengthened the ubiquitin bands (Fig. 3A). Generally, there are three types of ubiquitination modification of substrate proteins: a single ubiquitin molecule labeling a single lysine site (single monoubiquitination), multiple ubiquitin molecules labeling a single lysine site (poly monoubiquitination), and ubiquitin chains labeling multiple different lysine residues (polyubiquitination) [30]. If a particular protein has been extensively polyubiquitinated before it is recognized by the proteasome, then the application of lysine-less ubiquitin (UB‐K0) will reduce proteolysis [31]. As shown in Fig. 3B, co-expression with UB-K0 increased more than 1.35-fold in CPNE1 protein levels, indicating extensive polyubiquitination before CPNE1 degradation by the proteasome. To further identify the specific lysine residues of CPNE1 to which the ubiquitination chain is conjugated, we input the CPNE1 protein sequence from the NCBI database into different ubiquitin site prediction software programs (Table 1). K60, K84, K120, K135, K157, K190, K522, and K529 are the most likely ubiquitinated lysine residues, as predicted by several prediction websites. Then, we generated a set of CPNE1 mutants by substituting individual lysine (K) residues with arginine (R) residues (Fig. S1B). We found that the CPNE1 K157R mutant had a lower level of ubiquitination than WT CPNE1 (Fig. 3C). CPNE1 protein sequences of different species were downloaded from the NCBI database, and the CPNE1 K157 residue was found to be conserved across those species (Fig. 3D). Collectively, these results indicate that K157 is a major ubiquitination site of CPNE1.

**Table 1:** Prediction of CPNE1 ubiquitination sites.

Positions of predicted sites	Prediction tool	Web address of prediction tool
20,60,117,120,151,152,248,252,253,256,264,522,529	BDM-PUB	http://bdmpub.biocuckoo.org/result.php
60,73,84,135,157,182,190,522,529	UbiBrowser	http://ubibrowser.ncpsb.org
20,25,60,73,84,117,120,135,151,152,157,171,182,190,248,251,252,253,256,264,334,522,529	UbPred	http://www.ubpred.org/
25,84,120,135,157,190	UbiProber	http://bioinfo.ncu.edu.cn/ubiprober.aspx

### NEDD4L was identified as ubiquitin ligase for CPNE1

**Fig. 4:**
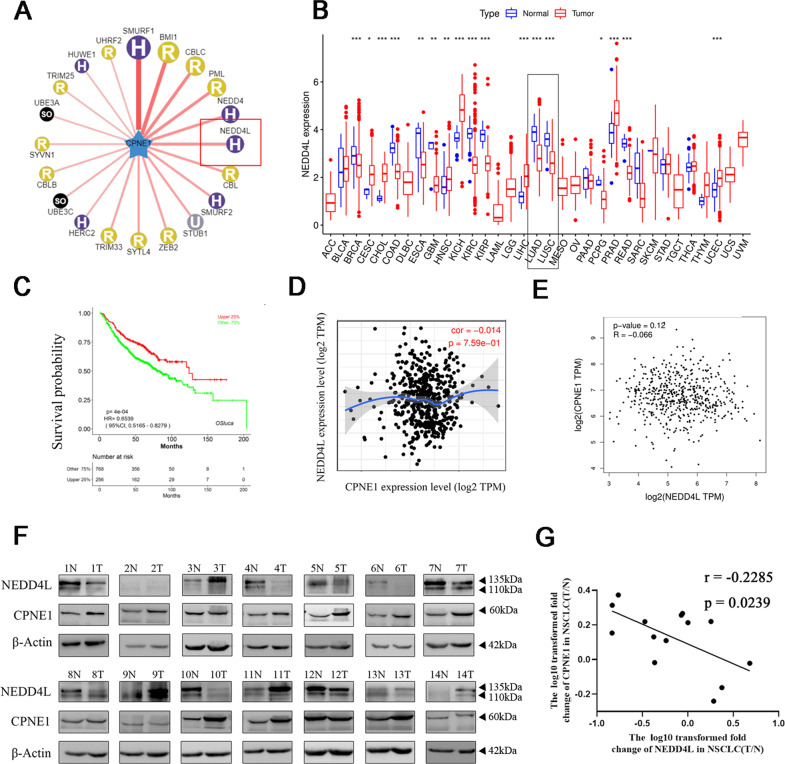
NEDD4L was identified as ubiquitin ligase for CPNE1. **A** Network view of the predicted E3 ligases for CPNE1 from Ubi-Browser. **B** NEDD4L expression is downregulated in multiple types of human cancer. **C** Low-level expression of NEDD4L is correlated with poor prognosis in lung cancer. **D**, **E** The mRNA levels of CPNE1 and NEDD4L are unrelated in non-small cell lung cancer. **F** Western blot analysis of CPNE1 and NEDD4L protein levels in 14 randomly selected NSCLC tissues and paired noncancerous lung tissues. The band density ratios represent the relative expression levels of CPNE1 and NEDD4L. **G** Relative protein expression levels of CPNE1 and NEDD4L in 14 paired NSCLC tissues. The *Y*-axis indicates the log10 transformed fold change in the T/N protein expression ratios of CPNE1 and NEDD4L. The number of each specimen is indicated below the *X*-axis.

E3 ligases often determine the specificity of ubiquitin-proteasome protein degradation. We next sought to identify the E3 ubiquitin ligase that targets CPNE1. NEDD4L was identified as the E3 ligase of CPNE1 via UbiBrowser (Fig. 4A). NEDD4L expression is downregulated in multiple types of human cancers (https://portal.gdc.cancer.gov) (Fig. 4B), suggesting that NEDD4L expression levels are downregulated in human lung cancer tissues and correlate with poor prognosis in lung cancer, in turn suggesting that NEDD4L deficiency may promote cancer cell invasion during malignant progression (http://bioinfo.henu.edu.cn/LUCA/LUCAList.jsp) (Fig. 4C). The results shown in Fig. 4D (http://gepia2.cancer-pku.cn) and Fig. 4E (https://cistrome.shi nyapps.io/timer/) confirm that there is no correlation between CPNE1 and NEDD4L at the mRNA level. We evaluated the expression levels of CPNE1 and NEDD4L in lung tissues (tumor specimens and adjacent non-tumor specimens) by Western blotting. Among 14 paired cancer tissues, negative or low protein expression of CPNE1 was detected in 4 samples (Fig. 4F, Table 2), and high expression was detected in 10 samples, while 57% were negative for NEDD4L expression and the rest were positive. There was a negative correlation between CPNE1 and NEDD4L at the protein level (Fig. 4G), which revealed that the interaction between these proteins occurs at the post-translational level. Taken together, these results indicate that NEDD4L plays a critical role in negative regulation of CPNE1.

**Table 2:** Demographic and clinical characteristics of patients and levels of CPNE1 protein expression in NSCLC tissue.

Characteristics	*n* = 14	CPNE1 protein expression high (*n* = 10) low (*n* = 4)		*P* value
Age (years)				
≤60	5 (35.7%)	2	3	0.095
>60	9 (64.3%)	8	1	
Gender				
Male	9 (64.3%)	6	3	0.545
Female	5 (35.7%)	4	1	
Histological features				
Adenocarcinoma	12 (85.7%)	9	3	0.505
Squamous cell carcinoma	2 (14.3%)	1	1	
Smoker				
Yes	9 (64.3%)	7	2	0.706
No	5 (35.7%)	3	2	
Clinical stage				
I +II	5 (35.7%)	4	1	0.545
III + IV	9 (64.3%)	6	3	
Distant metastasis				
No	12 (85.7%)	10	2	/
Yes	2 (14.3%)	0	2	
Lymph node metastasis				
No	5 (35.7%)	4	1	0.545
Yes	9 (64.3%)	6	3	

Data are presented as the mean ± SD values. The Kruskal–Wallis test was used for comparisons among three or more groups.

### NEDD4L interacts with the CPNE1 protein

**Fig. 5:**
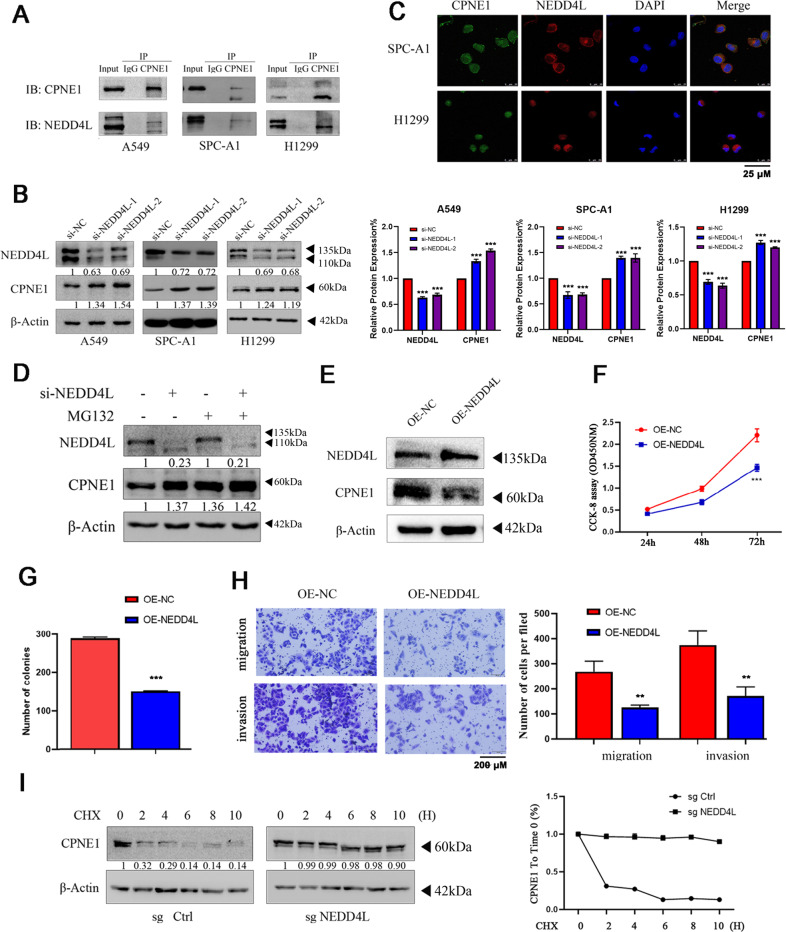
NEDD4L interacts with the CPNE1 protein. **A** The co-immunoprecipitation assay proved the association between endogenous CPNE1 and NEDD4L. Co-immunoprecipitation was performed as indicated. **B** CPNE1 protein levels in si-NEDD4L NSCLC cells. **C** Immunofluorescence was used to detect the intracellular localization of CPNE1 and NEDD4L. The intracellular localization of CPNE1 (green) and NEDD4L(red) is shown. **D** Upregulation of CPNE induced by NEDD4L interference was reduced by MG132 treatment. A549 cells were transfected with si-NEDD4L or si-Control. MG132 was added 6 h prior to protein extraction. **E** NEDD4L protein levels in NEDD4L overexpression A549 cells. **F** CCK-8 assay of cell viability in A549 (OE-NEDD4L compared with OE-NC). **G** the results of clonogenic analysis of cell proliferation in A549 cells (OE-NEDD4L compared with OE-NC). **H** Representative images of the transwell assay results for cell migration and invasion in A549 cells (OE-NEDD4L compared with OE-NC). **I** NEDD4L decreases CPNE1 half-life in HEK 293 T cells. Cells were transfected with sg-Control and sg-NEDD4L. 100 μg/ml cycloheximide was added for indicated times prior to protein extraction. Image J was used to quantify the CPNE1 protein level. **P < 0.01; ***P < 0.001.

To investigate the interaction between CPNE1 and NEDD4L, we performed a co-immunoprecipitation (co-IP) assay. CPNE1 co-immunoprecipitated with NEDD4L in A549, H1299, and SPC-A1 cells (Fig. 5A). We further investigated the localization of CPNE1 and NEDD4L in lung cancer cells. Immunostaining showed that NEDD4Lwas mainly localized in the cytosol, while CPNE1 was localized in both the cytosol and the nucleus (Fig. 5C). Next, we verified the regulatory effect of NEDD4L on CPNE1. NEDD4L downregulation increased CPNE1 protein expression (Fig. 5B). Interestingly, this increase was strongly suppressed by MG132, suggesting that the regulation of CPNE1 by NEDD4L is mediated via the ubiquitin-proteasome pathway (Fig. 5D). We established stable NEDD4L overexpression NSCLC cell lines; this was confirmed by the barely expressed level of NEDD4L protein expression in OE-NEDD4L A549 cells compared to that in vector transfected cells (OE-NC A549 cells) (Fig. 5E). The CCK-8 assay showed that cell proliferation was significantly inhibited in cells with overexpression of NEDD4L compared with the control cells at 24, 48, and 72 h (Fig. 5F). We also confirmed these findings by performing a clonogenic assay (Fig. 5G). Transwell assay of the A549 cells lines further indicated that overexpression of NEDD4L considerably suppressed the migration and invasion abilities of NSCLC cells (Fig. 5H). These results indicated that NEDD4L can suppress cell proliferation in NSCLC cells. CHX is widely used to inhibit protein synthesis in HEK 293 T cells. Following treatment with CHX, the sg-NEDD4L cells showed a significant increase in the half-life of CPNE1 suggesting that the regulation of CPNE1 by NEDD4L is mediated via the ubiquitin-proteasome pathway (Fig. 5I).

### NEDD4L knockout promotes tumor cell proliferation and metastasis through CPNE1

**Fig. 6:**
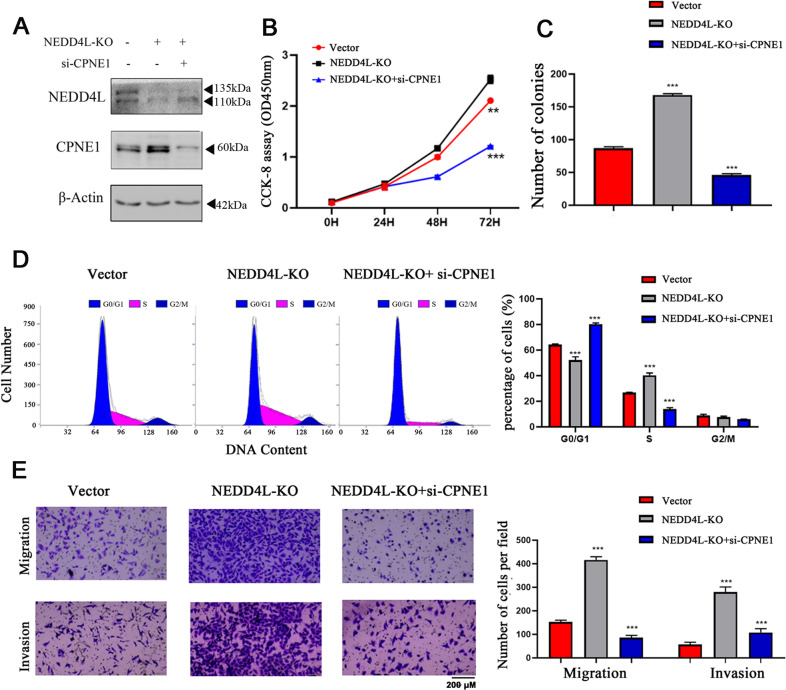
NEDD4L knockout promotes tumor cell proliferation and metastasis through CPNE1. **A** CPNE1 protein levels in NEDD4L-KO/NEDD4L-KO NSCLC cells and NSCLC cells with CPNE1 interference. **B** CCK-8 assay of cell viability in A549 cells. **C** Representative images showing the results of clonogenic assays of cell proliferation in A549 cells. Bar charts showing the colony formation of cells. **D** Flow cytometric assay of A549 cells. Cells were harvested 72 h after transfection and stained with PI. The percentage of cells in each cell cycle phase is shown in the inset of each panel. **E** Representative images showing the results of transwell cell migration and invasion assays in A549 cells. **P < 0.01; ***P < 0.001.

To further characterize the regulation of NEDD4L-induced CPNE1 upregulation in lung cancer cells, we established stable NEDD4L knockout (NEDD4L KO) A549 cell lines (Fig. 6A). NEDD4L-KO enhanced the proliferation of A549 cells, whereas CPNE1 interference reversed this facilitatory effect (Fig. 6B, C). Subsequently, the proportion of NEDD4L-KO cells in G0/G1 phase and the proportion of NEDD4L-KO cells in S phase were significantly lower and higher, respectively, than the corresponding proportions of control cells. Similarly, this effect was inhibited by interference with CPNE1 expression (Fig. 6D). Transwell migration and invasion assays showed that NEDD4L knockout promoted lung cancer cell migration and invasion. Furthermore, the migration and invasion abilities were restored by CPNE1 interference (Fig. 6E). In conclusion, the regulatory activity of NEDD4L on CPNE1 can affect the biological function of CPNE1.

### Suppression of in vivo tumor growth by NEDD4L overexpression

**Fig. 7:**
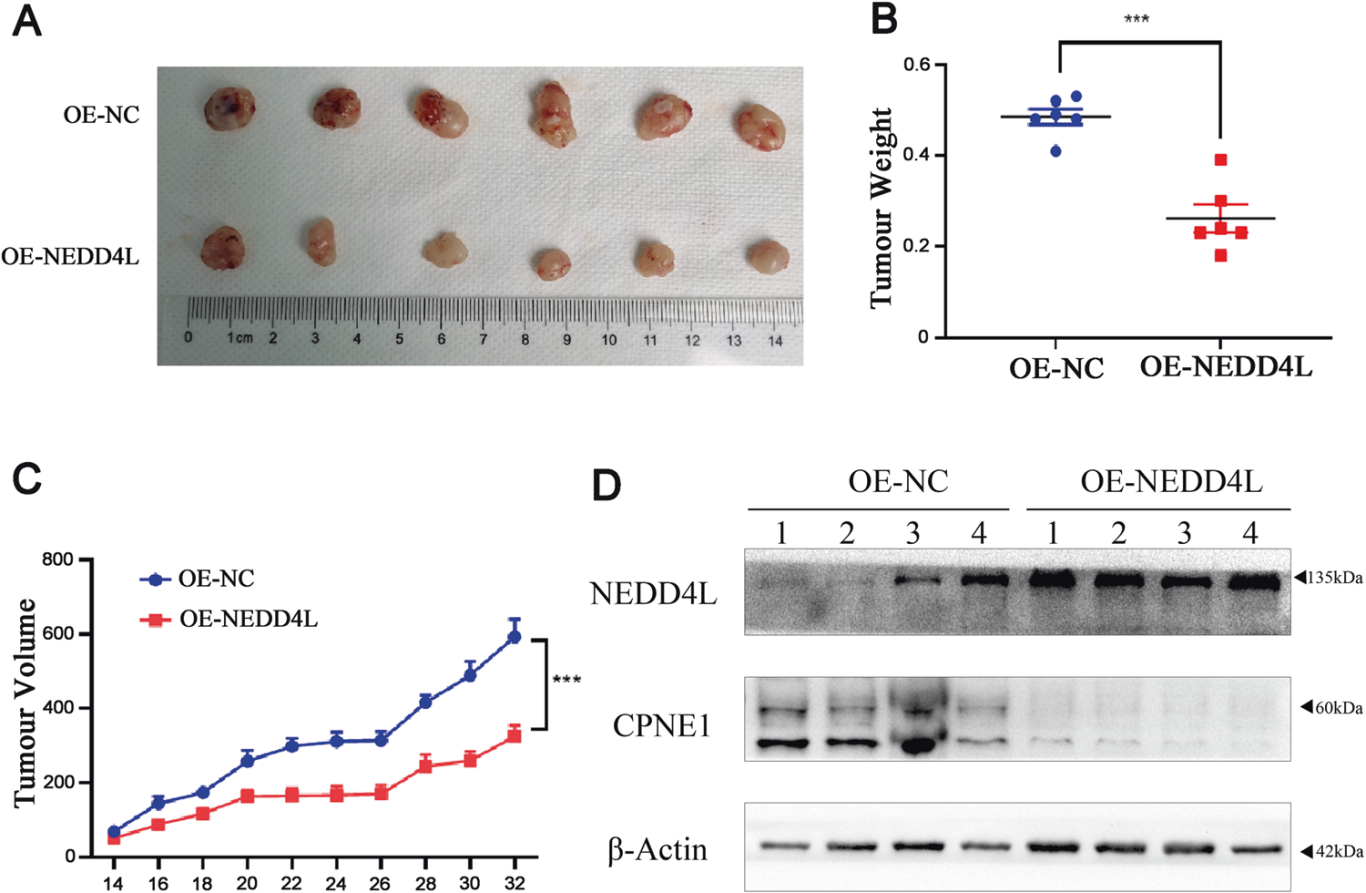
Suppression of in vivo tumor growth by NEDD4L overexpression. **A** NEDD4L overexpression in A549 cell xenografts in nude mice (*n* = 6) at the experimental endpoint; tumors were dissected and photographed as shown. **B** Each tumor was weighed. **C** Tumor growth curves in mice (*n* = 6 in each group) inoculated with the indicated cells at the indicated days. **D** NEDD4L protein expression in tumors was detected by Western blot. ***P < 0.001.

To further assess the in vivo effect of NEDD4L overexpression on NSCLC cells, A549 cells with stable NEDD4L overexpression were inoculated into BALB/C athymic mice. As shown in Fig. 7A, C, tumors formed by the cells with NEDD4L overexpression were much smaller in size than those formed from the control cells. In line with these results, tumor weight was found to be lower in cells with NEDD4L overexpression (Fig. 7B). The tissues resected from the xenograft tumors were analyzed to verify NEDD4L and CPNE1 expression using Western blot (Fig. 7D).

## Discussion

Our previous studies confirmed that CPNE1 plays an important role in the development of cancer. CPNE1 was highly expressed in lung cancer and was positively correlated with TNM stage and lymph node metastasis. Overexpression of CPNE1 can activate Src, FAK, AKT, ERK, and other signaling pathways in vivo and promote the proliferation and metastasis of lung cancer cells. To elucidate the specific mechanism of the high expression of CPNE1 in lung cancer, we studied its regulation at the pre-transcriptional level. We found that miR-335-5p inhibited the expression of CPNE1 by directly targeting the CPNE13′-UTR, thus inhibiting the proliferation and invasion of NSCLC cells. In addition, miR-195-5p is responsible for the ability of high CPNE1 expression to result in poor prognosis in squamous cell lung cancer (SCC) and lung adenocarcinoma (ADC).

**Fig. 8:**
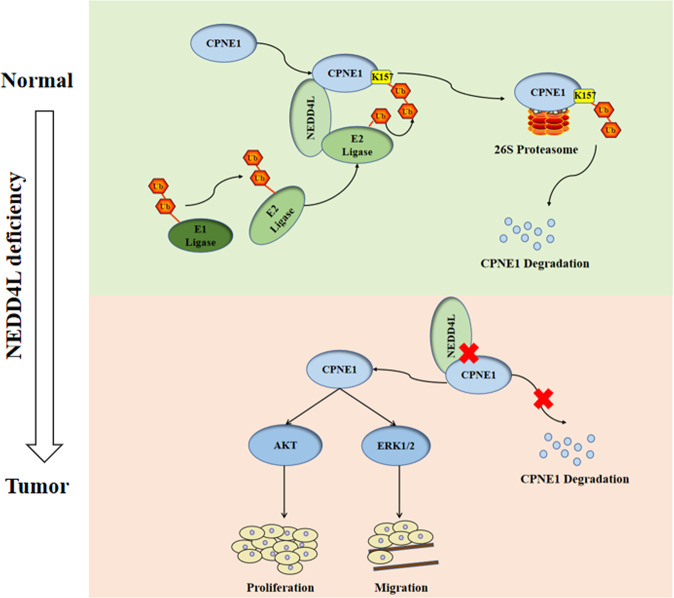
The hypothetical model for the functional interplay of CPNE1 with NEDD4L in non-small-cell lung cancer cells. A merchanism working model of NEDD4L regulates the CPNE1.

This study was the first to investigate the mechanism of CPNE1 degradation. We demonstrated that CPNE1 can be degraded by both the ubiquitin-proteasome and lysosomal proteolysis pathways. This study mainly focused on the degradation of CPNE1 via the ubiquitin-proteasome pathway. First, we confirmed that CPNE1 can interact with ubiquitin and that the CPNE1-K157 residue played a significant role in this interaction. Second, we identified NEDD4L as a ubiquitin ligase for CPNE1. NEDD4L knockout stabilized the CPNE1 protein and enhanced the proliferation and metastasis of lung cancer cells. In addition, the effect of NEDD4L was reversed by CPNE1 interference (Fig. 8). Additional studies are needed to further validate our hypothesis. The K157 residue of CPNE1 is essential for the ubiquitination of CPNE1, but whether it can affect the biological function of CPNE1 by affecting the ubiquitination of CPNE1 needs further study. Residues subject to polyubiquitination modification include K6, K11, K27, K29, K33, K48, and K63. Polyubiquitination at K11 and K48 mainly plays a role in protein degradation and the regulation of protein stability. Modification of K63 mainly affects signal transduction, DNA repair, and regulation of protein activity. Modification of K6 is related to mitosis [32]. It is important to determine the amino acid residues involved in the interaction between Ub and CPNE1. In addition, the mechanism by which NEDD4L regulates CPNE1 remains to be elucidated. For example, the domain via which CPNE1 and NEDD4L interact is unknown.

In conclusion, our study is the first to report the specific mechanism of CPNE1 degradation and showed that NEDD4L is responsible for CPNE1 degradation by the ubiquitin-proteasome pathway. NEDD4L KO can stabilize the CPNE1 protein and inhibit the proliferation and metastasis of NSCLC cells. In addition, we found that CPNE1 interacted with Ub via its K157 residue. Therefore, our findings further reveal the specific mechanism of CPNE1 in NSCLC carcinogenesis. The discovery of this NEDD4L-mediated ubiquitination of CPNE1 provides new insight into therapeutic strategies for NSCLC.

## Materials and methods

### Tissue samples

Paired NSCLC and adjacent noncancerous lung tissue samples were collected with the informed consent of patients from the First Affiliated Hospital of Soochow University. The patients had been diagnosed with NSCLC based on their histological and pathological characteristics according to the revised International System for Staging Lung Cancer. They had not undergone chemotherapy or radiotherapy before tissue sampling. The tissue samples were snap frozen and stored in a cryogenic freezer at −80 °C. This study was approved by the Academic Advisory Board of Soochow University.

### Cell culture

The human NSCLC cell lines A549, H1299, SPC-A1, and HCC827 and HEK293T cells were purchased from the Cell Bank of the Chinese Academy of Sciences (Shanghai, China). Cells were cultured in RPMI 1640 medium and DMEM supplemented with 10% fetal bovine serum (Gibco, Carlsbad, CA, USA) and L-glutamine (Invitrogen, Carlsbad, CA, USA) under standard cell culture conditions (5% CO_2_/37 °C).

### Plasmid construction and mutagenesis

The CDS region of CPNE1 was amplified using cDNA from H1299 cells as the template and was cloned into FLAG-PCDH. For mutagenesis, Flag-CPNE1 was used as the template for PCR amplification. Amplification products were mixed with 2 µl of buffer and 1 µl of DpnI restriction enzyme and incubated at 37 °C for 3 h. Then, the mixture was used for bacterial transformation, and sequencing was then conducted at Suzhou GENEWIZ Biotechnology. The primer sequences used for the construction of Flag-CPNE1 and mutagenesis are shown in Table 3.

**Table 3:** Primer sequences used for the introduction of Flag-CPNE1 mutations.

Mutations	Primer sequences
K60R	F:AAGCCCTGAGTTCTCCAGGACTCTACAGCTTGAGT R:ACTCAAGCTGTAGAGTCCTGGAGAACTCAGGGCTT
K84R	F:CTATGACATAGACAACAGGACGCCAGAGCTGAGGG R:CCCTCAGCTCTGGCGTCCTGTTGTCTATGTCATAG
K120R	F:GATGCTGAAGCCTGGAAGGCCTGCTGGGCGGGGGA R:TCCCCCGCCCAGCAGGCCTTCCAGGCTTCAGCATC
K135R	F:CTCAGCTCAGGAATTAAGGGACAATCGTGTAGTAA R:TTACTACACGATTGTCCCTTAATTCCTGAGCTGAG
K157R	F:GAAGGACTTCCTGGGAAGGTCAGATCCATTTCTGG R:CCAGAAATGGATCTGACCTTCCCAGGAAGTCCTTC
K190R	F:CCTGAACCCTACATGGAGGCGTTTCTCAGTCCCCGT R:ACGGGGACTGAGAAACGCCTCCATGTAGGGTTCAGG
K522R	F:AGGGTTGGGCCCCGCTCAGGCCACTTCCACCCTCAG R:CTGAGGGTGGAAGTGGCCTGAGCGGGGCCCAACCCT
K529R	F:ACTTCCACCCTCAGCCAGGGATCCTGCACAGGCC R:GGCCTGTGCAGGATCCCTGGCTGAGGGTGGAAGT

### Protein stability assays

CHX, an inhibitor of protein synthesis, was used to determine the half-life of CPNE1. Cells were treated with CHX (100 μg/ml) for 2, 4, 6, 8, and 10 h separately before protein extraction. CPNE1 degradation was analyzed by Western blotting.

### RNA interference

Two pre-designed small interfering RNA (siRNA) sequences corresponding to the target sequences were directly synthesized (GenePharma). The siRNA constructs are described as follows: siRNA-NEDD4L-1: 5′-GAGTACCTATGAATGGATT-3′(sense) and 5′-AATCCATTCATAGGTACTC-3′(antisense); siRNA-NEDD4L-2: 5′-CAGAAATAATGGTCACAAA-3′(sense) and 5′-TTTGTGACCATTATTTCTG-3′ (antisense); siRNA-CPNE1-1: 5′-GCAGGUCUCGCAU GAAUUUTT-3′(sense) and 5′-AAAUUCAUGCGAGACCUGCTT-3′ (antisense). Transfection of siRNA into cells was performed with Lipofectamine 2000 according to the instructions of the manufacturer.

### Western blot analysis and co-immunoprecipitation assay

Western blotting (WB) was performed as previously described [33]. The antibodies used for the analysis were anti-CPNE1 (Abcam, Shanghai, China) and anti-NEDD4L (CST, UK). For co-immunoprecipitation experiments, 2 × 10^5^ HEK293T cells were transfected with the required plasmids for 48 h. Cells were harvested with 1 ml of RIPA buffer. Equal amounts of protein were incubated with 2 μg of M2 anti-Flag affinity agarose at 4 °C for 4 h with end-over-end rotation. The protein–antibody complexes were collected by centrifugation and washed 3 times with RIPA buffer. Then, the precipitates were analyzed by Western blotting.

### Immunofluorescence

In brief, cells were fixed with 4% formaldehyde for 15 min and blocked with 5% BSA for 30 min. Then, the cell climbing slices were incubated overnight with a rat anti-NEDD4L antibody (1:100; CST, UK) and a mouse anti-CPNE1 antibody (1:100; Santa, Temecula, CA). After washing with PBS three times, the cell climbing slices were incubated with a fluorescent secondary antibody at room temperature for 1 h. Nuclei were then stained with DAPI. Finally, the slices were observed and images were acquired under a confocal microscope.

### Cell proliferation analysis

Cell proliferation was evaluated using CCK-8 assays. Cells were digested and plated at a concentration of 3 × 10^3^ cells per well in 96-well plates under standard cell culture conditions. A Cell Counting Kit-8 (Boster, Wuhan, China) was used to detect cell proliferation after culture for 24, 48, and 72 h.

### Colony formation assay

A colony formation assay was used to confirm the malignant transformation. Three thousand cells were seeded in 3 ml of RPMI 1640 medium supplemented with 10% fetal bovine serum and incubated at 37 °C with 5% CO_2_. The number of colonies formed after 14 days was counted using ImageJ.

### Migration and invasion assays

Cell motility was assayed using 12-well transwell plates (Corning) as described before [28]. Experiments were performed in 24-well transwell plates with 8-µm pore-size chambers. The difference between the cell migration and invasion assays was whether the top surface of the filter was pre-coated with Matrigel. In brief, lung cancer cells (5 × 10^4^) were plated in the upper compartment of the transwell chamber in medium containing 1% FBS. Then, 0.8 ml of complete medium was added to the lower compartment of the chamber. Cells on the upper surface of the filter were removed after 24 h in both the migration and invasion assays. Images were acquired using a microscope (CKX41, Olympus).

### Xenografts

BALB/c athymic nude mice (female, 4–6 weeks old, weighing 16–20 g) were purchased from the Experimental Animal Center of Soochow University and bred under pathogen-free conditions. All animal experiments were carried out following the Guide for the Care and Use of Experimental Animals of the Experimental Animal Center of Soochow University. Three million stable NEDD4L cells (A549-overexpression-NC cells and A549-overexpression-NEDD4L cells) were suspended in 150 μl of FBS-free medium and subcutaneously injected into the nude mice, which were randomly divided into two groups (6 mice per group). Tumor growth was analyzed by measuring the tumor length (*L*) and width (*W*) and calculating the volume (*V*) with the formula *V* = *LW*^2^/2.

### Statistical analysis

We used Student’s *t* test (two-tailed) and two-way ANOVA to analyze the results when required. *P* < 0.05 indicates that a difference between groups was significant. All data were analyzed using GraphPad Prism 8.

## Supplementary information

Figure legend of supplementary figure

Supplementary Figure1

Authorship

## Data Availability

The datasets used and analyzed in this study are available from the corresponding author on reasonable request.
