# Supplementary material for: The regulation of CPNE1 ubiquitination by the NEDD4L is involved in the pathogenesis of non-small cell lung cancer
Source: Cell Death Discov. 2021 Nov 6;7:336. doi: 10.1038/s41420-021-00736-1 (PMC8572224; doi:10.1038/s41420-021-00736-1)
Supplement: Supplementary file 1 — Figure legend of supplementary figure [file 41420_2021_736_MOESM1_ESM.docx]

**Fig. S1** (A) CPNE1 is an oncogene in the OSluca database. (B) Construction of CPNE1 mutation plasmid by substituting individual lysine (K) residues with arginine (R) residues.
