# Supplementary material for: The regulation of CPNE1 ubiquitination by the NEDD4L is involved in the pathogenesis of non-small cell lung cancer
Source: Cell Death Discov. 2021 Nov 6;7:336. doi: 10.1038/s41420-021-00736-1 (PMC8572224; doi:10.1038/s41420-021-00736-1)
Supplement: Supplementary file 3 — Authorship [file 41420_2021_736_MOESM3_ESM.pdf]

DECLARATION OF CONTRIBUTIONS TO ARTICLE

ADMC

Manuscript Number:	Journal Name:	
CDDISCOVERY-21-2097	*Cell Death Discovery*	(the 'Journal')

Proposed Title of the Contribution:

The regulation of CPNE1 ubiquitination by the NEDD4L is involved in the pathogenesis of non-small cell lung cancer (the 'Contribution)

Author(s):

Ruochen Zhang, Weijie Zhang, Yuanyuan Zeng, Yue Li, Jieqi Zhou, Yang Zhang, Anqi Wang, Yantian Lv, Jianjie Zhu, Zeyi Liu, Jian-an Huang (the 'Authors')

For all *CDDis* articles, each person named as an author in the published version must be able to show he or she has contributed substantially to the article.

Authorship credit should be based on 1) substantial contributions to conception and design, acquisition of data, or analysis and interpretation of data; 2) drafting the article or revising it critically for important intellectual content; and 3) final approval of the version to be published. Authors should meet conditions 1, 2 and 3.

Any person who cannot be shown to have made a substantial contribution to the article cannot be listed as an author in the final version. The name of any person who is deemed to have made a minor contribution can, however, appear in the Acknowledgments section of the article.

Please complete the table below to indicate the contributions of all named authors to the manuscript.

Author Full Name:	Specification of Contribution to the Manuscript:
Ruochen Zhang	Performed the experiments, wrote the paper.
Weijie Zhang	Performed the experiments
Yuanyuan Zeng	Performed the experiments
Yue Li	Analyzed data and help conduct partial experiment
Jieqi Zhou	Analyzed data and help conduct partial experiment
Yang Zhang	Provided helpful discussion and reviewed the manuscript
Anqi Wang	Provided helpful discussion and reviewed the manuscript
Yantian Lv	Provided guidance on experimental technology and gave suggestions
Jianjie Zhu	Provided guidance on experimental technology and gave suggestions
Zeyi Liu	Revised the manuscript and supervised this study
Jian-an Huang	Revised the manuscript and supervised this study

Please complete the table below to indicate the contributions of all named authors to the figures.

Figure 1:

Ruochen Zhang, Weijie Zhang, Yue Li performed the data analysis.

Figure 2:

Ruochen Zhang and Weijie Zhang generated the western blot data. Ruochen Zhang and Yuanyuan Zeng performed the data analysis.

Figure 3:

Ruochen Zhang, Weijie Zhang, and Yuanyuan Zeng generated the western blot data. Yuanyuan Zeng construct the plasmid. Jieqi Zhou and Yue Li performed the data analysis.

Figure 4:

Ruochen Zhang and Weijie Zhang generated the western blot data. Yuanyuan Zeng performed the data analysis. Anqi Wang and Yantian Lv provided guidance on experimental technology and gave suggestions.

Figure 5:

Ruochen Zhang, Weijie Zhang, Yuanyuan Zeng performed the western blot data. Ruochen Zhang construct the plasmid. Weijie Zhang and Jieqi Zhou performed the cck-8, transwell, and immunofluorescence.Anqi Wang and Yang Zhang performed the data analysis.

Figure 6:

Ruochen Zhang, Weijie Zhang, Yuanyuan Zeng performed the western blot data. Ruochen Zhang and Yuanyuan Zeng construct the A549 cells with stable NEDD4L knockout. Weijie Zhang performed the cck-8, transwell assay. Yue Li and Yang Zhang performed the data analysis.

Signed for and on behalf of the Author(s):	Print Name:	Date:
Zeyi Liu	Zeyi Liu	2021-08-30

Please complete the table below to indicate the contributions of all named authors to the figures.

Figure 7:

Ruochen Zhang and Weijie Zhang generated all data in vivo mouse model. Yue Li and Jieqi Zhou performed the data analysis.

Supplementary Figure1:

The Flag-CPNE1 and mutagenesis was constructed by Zhang Ruochen and Yuanyuan Zeng.

Supplementary Figure2:

Supplementary Figure3:

Supplementary Figure4:

Supplementary Figure7:

Signed for and on behalf of the Author(s):	Print Name:	Date:
Zeyi Liu	Zeyi Liu	2021-08-30
